# Supplementary material for: Evidence Based on an Integrative Analysis of Multi-Omics Data on METTL7A as a Molecular Marker in Pan-Cancer
Source: Biomolecules. 2023 Jan 18;13(2):195. doi: 10.3390/biom13020195 (PMC9952925; doi:10.3390/biom13020195)
Supplement: Supplementary file 1 [file biomolecules-13-00195-s001.zip › biomolecules-2077066-supplementary.pdf]

Supplementary Figures

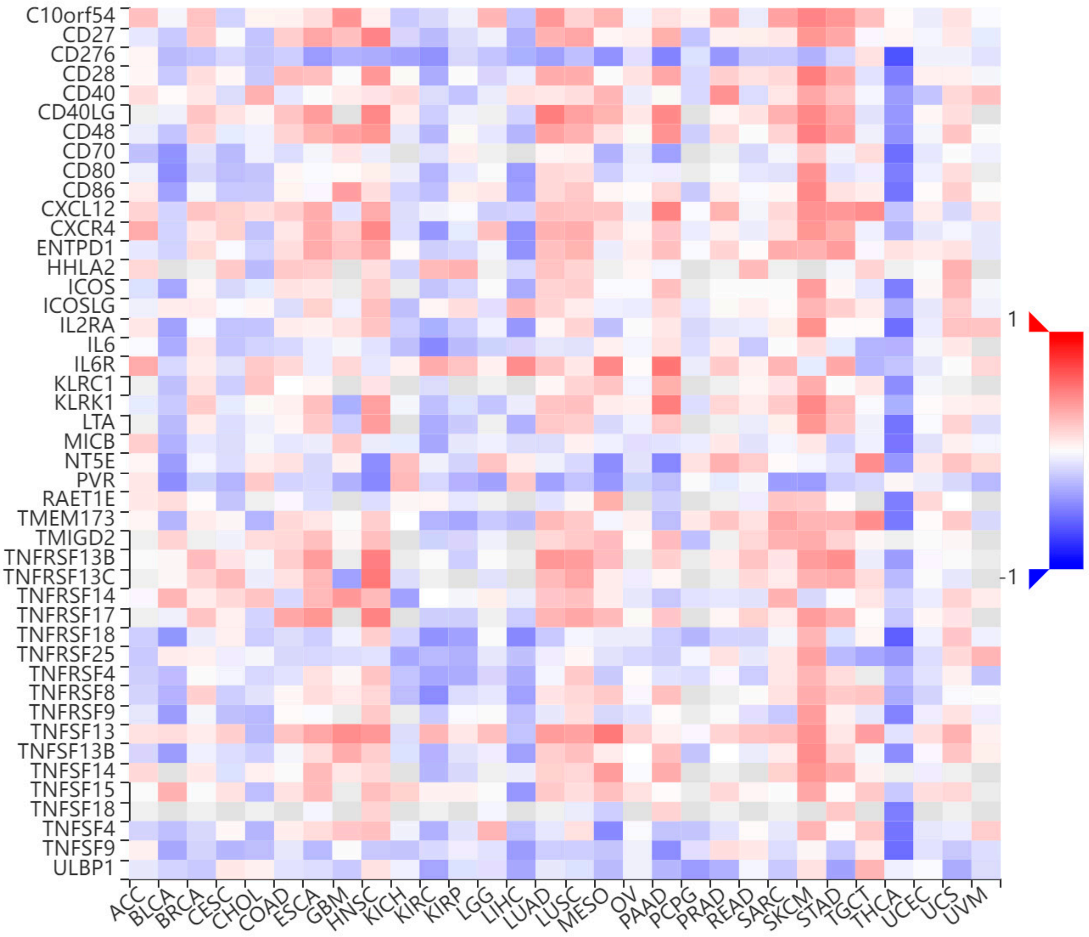

Supplementary Figure S1: Correlation of the METTL7A expression with immune stimulators in malignant tumors.

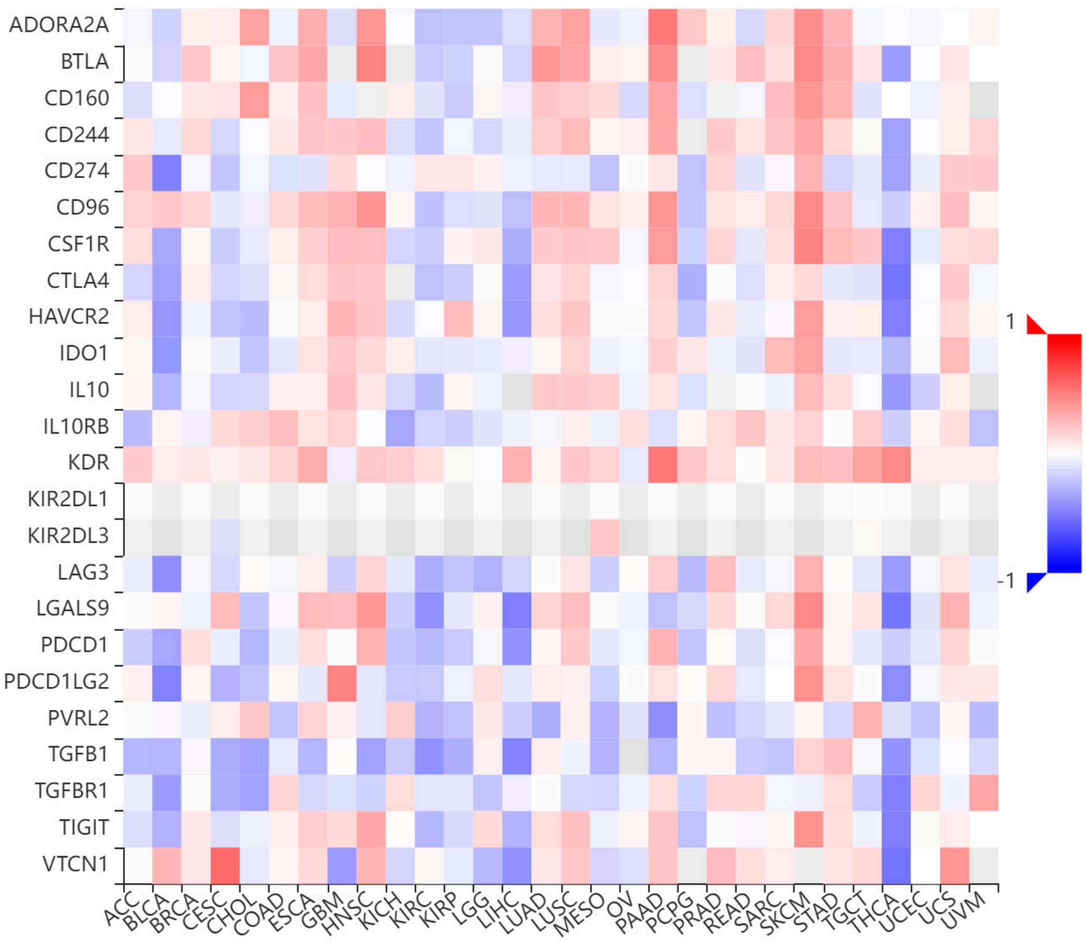

Supplementary Figure S2: Correlation of the METTL7A expression with immune inhibitors in malignant tumors.

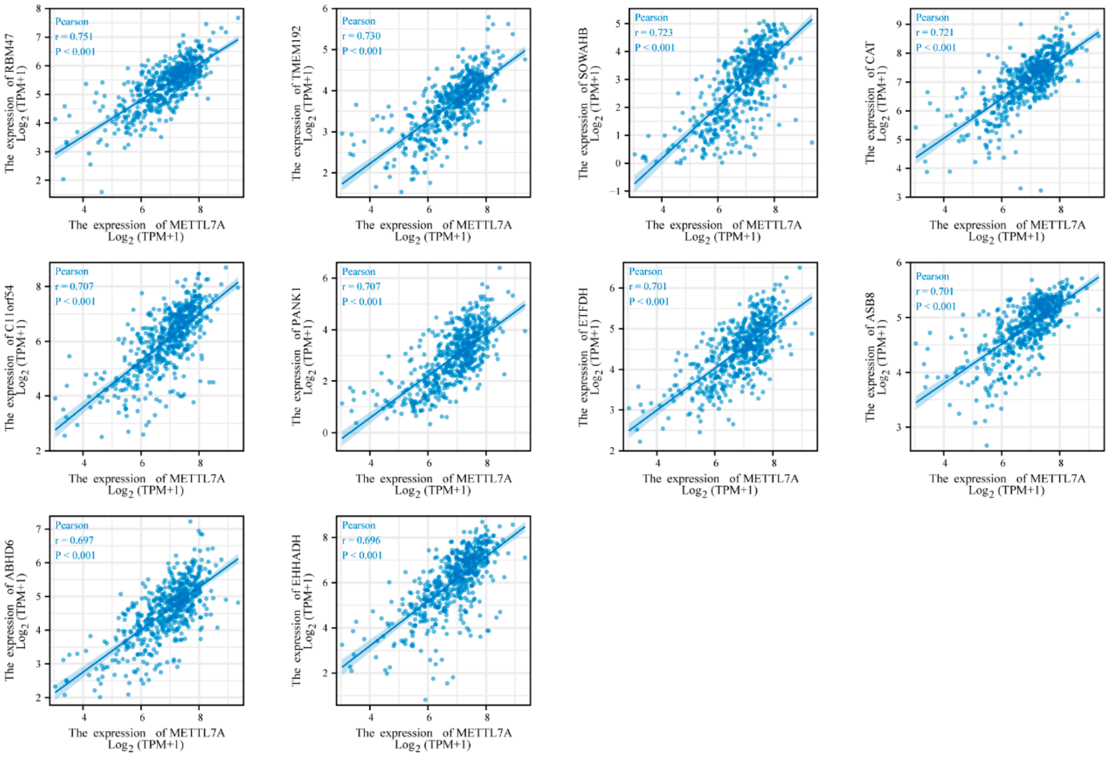

Supplementary Figure S3: Correlation between the expression of METTL7A and the expression of the top 10 positively co-expression genes.

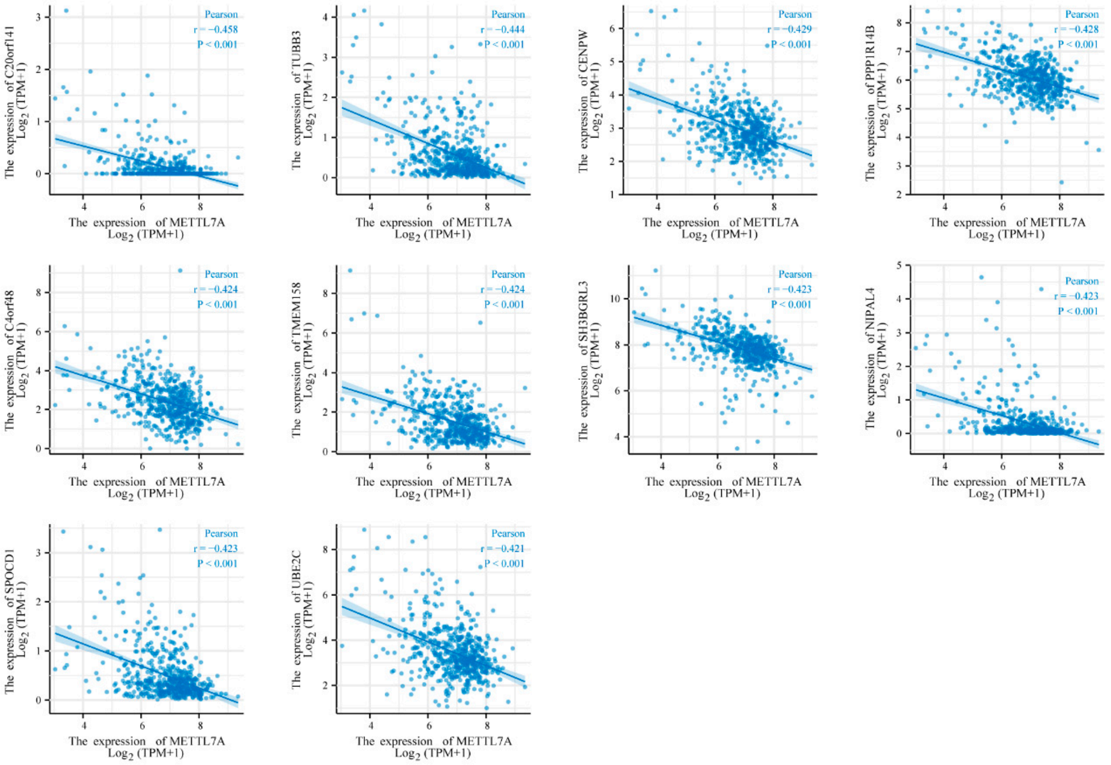

Supplementary Figure S4: Correlation between the expression of METTL7A and the expression of the top 10 negatively co-expression genes.

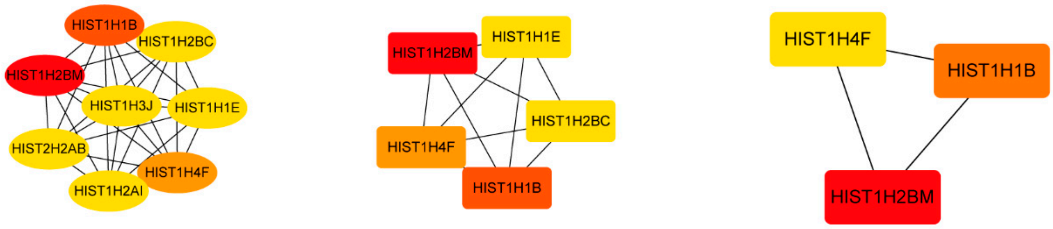

Supplementary Figure S5: The hub genes of protein-protein interaction (PPI) network of DEGs.

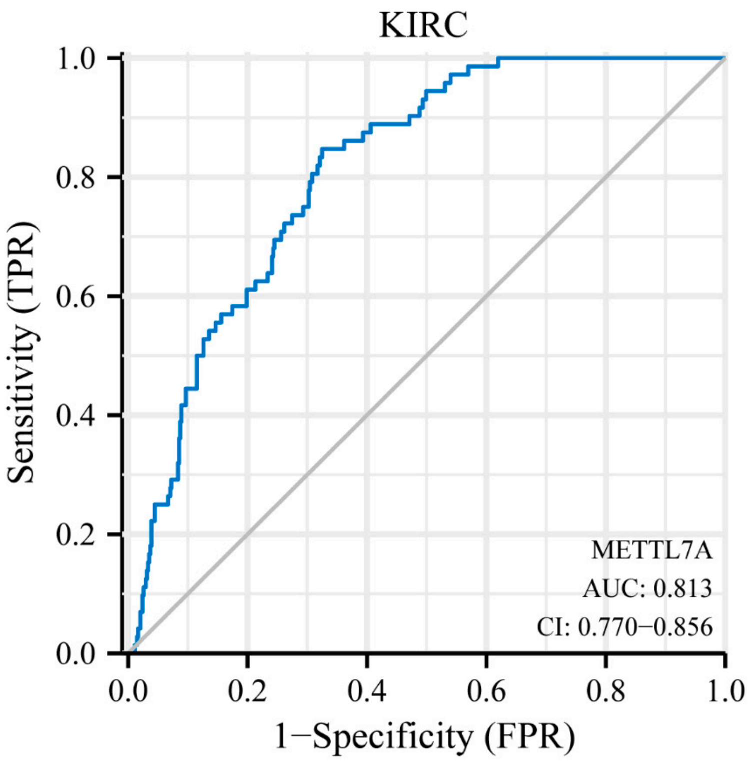

Supplementary Figure S6: Receiver operating characteristic (ROC) curve for METTL7A expression in KIRC.
